# The Importance of a Late First Trimester Placental Sonogram in Patients at Risk of Abnormal Placentation

**DOI:** 10.1155/2014/345348

**Published:** 2014-06-01

**Authors:** Felipe Moretti, Maria Merziotis, Zachary M. Ferraro, Lawrence Oppenheimer, Karen Fung Kee Fung

**Affiliations:** Obstetrics, Gynecology and Newborn Care, Division of Maternal-Fetal Medicine, University of Ottawa, The Ottawa Hospital, General Campus, 501 Smyth Road, Ottawa, ON, Canada K1H 8L6

## Abstract

*Background*. Placenta accreta is a potentially life-threatening obstetrical condition and is responsible for many emergency Caesarean hysterectomies. Early prenatal diagnosis may help minimize maternal morbidity and mortality. This report highlights risk factors, early diagnostic findings and complications associated with placenta accreta, and the role of first trimester sonography in diagnosis. *Case*. A 38-year-old pregnant woman, G2P1L1 with history of one previous Caesarean section, presented with vaginal bleeding at 13 weeks' gestation. Ultrasound examination was highly suspicious of placenta previa with accreta. During an earlier 12-week scan for nuchal translucency measurement, the placenta was suboptimally visualized. She was counselled regarding potential maternal and fetal complications as well as management options. At 33 weeks' gestation Caesarean hysterectomy was performed due to vaginal bleeding. *Conclusion*. Early ultrasound screening in high-risk patients may be advantageous in order to identify placenta accreta and conduct appropriate patient counseling regarding risks and management options.

## 1. Introduction

Placenta accreta is a potentially life-threatening obstetrical emergency. It occurs when there is abnormal adherence of the placenta to the uterine wall, involving a defect in the decidua basalis [1, 2]. The placenta invades the myometrium of the uterus (accreta), with more extensive invasion through the uterine serosa, ureters, bladder, and bowel in placenta percreta/increta [1, 3]. According to Miller et al. [4], the most common abnormal attachment is placenta accreta. This superficial invasion represents 75% of cases. Placenta increta (invasion deeper into the myometrium) represents 18% of cases, and placenta percreta (invasion all the way through the uterus and into surrounding structures) represents 7% of cases. Consequently, there is incomplete separation of the placenta from the uterus after delivery leading to significant postpartum hemorrhage [1, 2]. Placenta accreta is commonly treated by hysterectomy in order to avoid excessive hemorrhage and control the bleeding [1, 3].

Clinical risk factors for placenta accreta include characteristics such as multiparity and age. Obstetrical risk factors include placenta previa, prior uterine surgery, and previous Cesarean section [1, 3, 5]. The incidence of placenta accreta ranges from 1/2510 to 1/533 pregnancies with an iatrogenic rise noted in developing countries in parallel with rising Caesarean section rates [1, 2, 5–7]. Current research suggests a 25%–50% incidence of placenta accreta in patients with a placenta previa and prior Cesarean delivery [4]. Thus, the clinical burden of placenta accreta continues to rise. Additionally, placenta accreta is responsible for 33%–50% of emergency peripartum hysterectomies [8–10]. Prenatal screening and diagnosis have been shown to help reduce the risk of various complications and blood loss [11, 12]. We believe that early prenatal diagnosis of placenta accreta may maximize treatment options, minimize maternal morbidity and mortality, and optimize presurgical planning, while improving obstetrical outcomes and patient well-being [1–3, 5]. The purpose of this report is to review the literature addressing first trimester diagnosis of placenta accreta and explore the relationship between maternal risk factors, first trimester diagnosis, and outcomes.

## 2. Case Presentation

**Figure 1:**
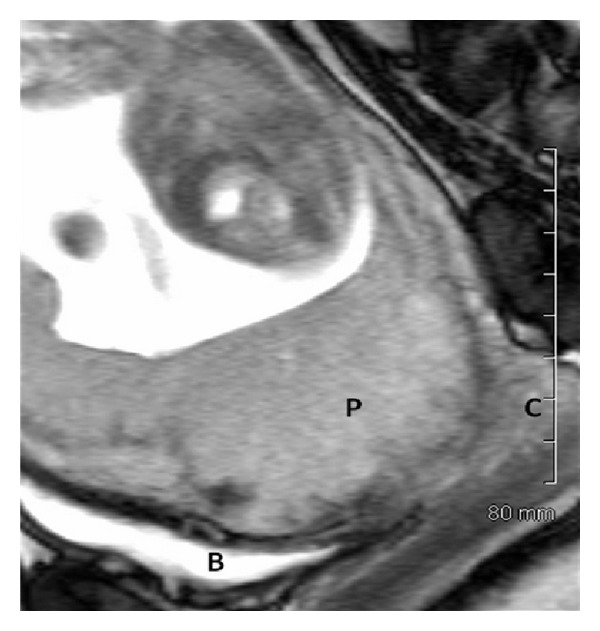
Magnetic resonance imaging (MRI) at 17 weeks' gestation demonstrating focal disruption of the anteroinferior myometrium with placental invasion. P: placenta, B: bladder, and C: cervix.

A 38-year-old pregnant woman, G2P1, with a history of previous Caesarean section, presented at 13 weeks of pregnancy with vaginal bleeding. Ultrasound examination demonstrated anterior placenta previa with loss of the retroplacental hypovascular clear zone or vascular plexus and turbulent vascular flow in irregular vascular spaces or lacunae, within the placenta, giving a high suspicion of accreta. A previous nuchal translucency scan was performed at an outside institution at 12 weeks' gestation without comment on the placental location or morphology. An MRI study at 17 weeks validated the sonographic impression of placenta accreta (Figure 1). She was referred to the regional tertiary care perinatal unit and counselled extensively regarding potential maternal and fetal complications and management options. The patient elected to continue with the pregnancy. At 33 weeks, her baby was delivered by emergency Caesarean section with Caesarean hysterectomy due to vaginal bleeding requiring 4 units of red blood cells. Placental pathology confirmed accreta. The patient was discharged on post-op day 4 and the neonate discharged on day 12.

## 3. Discussion

Placenta accreta is the most common placental attachment disorder found in approximately 75% of all cases of abnormally adherent placentas [4]. This disorder is thought to result from a deficiency in the decidua basalis layer of the uterus, leading to direct trophoblast invasion into the myometrium [13, 14]. Other etiologies, including a primary defect in trophoblast function or abnormal tissue oxygenation or vascularization of a deficient uterine scar, may also contribute to this disorder [15]. It is more commonly found in association with placenta previa, an independent risk factor, likely attributable to the relative dearth of decidua basalis in the lower uterine segment in comparison to the rest of the uterus [16]. Recently, Jauniaux and Jurkovic [15] reviewed potential causal factors leading to abnormal placentation in placenta accreta including a primary defect of trophoblast function, a secondary basalis defect due to a failure of normal decidualization, and abnormal vascularisation and tissue oxygenation of the scarred area. Other known risk factors include maternal age, uterine malformations, uterine fibroids, multiparity, corneal implantation site, previous uterine instrumentation, and surgery such as dilation and curettage (D&C), manual removal of placenta, and previous Caesarean section.

Placenta accreta remains the leading cause of peripartum hysterectomy [16] with a maternal mortality rate of approximately 6% due to complications of severe hemorrhage. Both early and late complications of this diagnosis occur resulting in maternal morbidity and mortality, including large volume blood loss requiring transfusion, prolonged admission to intensive care units, coagulopathy, and ureteral injury. Late complications include infection, hospital readmission, and multiple surgeries [17]. Other obstetrical complications including preterm birth and intrauterine growth restriction are also increased with this diagnosis [18].

Principles of management of this condition include extensive counseling of the patient regarding therapeutic options, either definitive (hysterectomy) or conservative (leaving placenta in situ), based on the patients' desire for future childbearing, setting realistic patient expectations of outcomes, and need for close followup (if conservative management chosen), presurgical planning, and multidisciplinary consultation (interventional radiology, anesthesia, neonatology, blood bank, etc.). Antepartum diagnosis of this condition is preferable and leads to improved patient outcomes. Following an intrapartum diagnosis of placenta accreta the need for maternal blood transfusion is in the order of 70% [19]. Antepartum diagnosis of placenta accreta reduces the need for large blood transfusions amounting to ≥4 units [20]. Similarly, antepartum diagnosis offers the advantage of conservative management, leaving the placenta in situ and maintaining reproductive opportunities. Overall, proper assessment of the placenta in early gestation may increase awareness of known risk factors and facilitate proper treatment. Knowledge of the key points discussed below is essential for early diagnosis of placenta accreta and proper counseling for complications and management.

Ultrasound is a primary screening tool in the diagnosis of placental attachment disorders. Although abnormal invasion may persist throughout gestation, ultrasound visualization may differ depending on the type of attachment disorder. Usually diagnosis of abnormal placental location or attachment is achieved at a second or third trimester imaging study. With wide acceptance of first trimester aneuploidy screening with ultrasound measurement of the fetal nuchal translucency, authors have reported diagnosis of placental disorders in the first trimester. Several ultrasound features have been documented in the literature to be associated with a high suspicion of placenta accreta. These include increased myometrial thickness, presence of placental lacunae, loss of the clear space between the placenta and myometrium, and anomalies of the interface of the bladder and myometrium [21]. A recent study by Ballas et al. [5] suggests that sonographic findings in the first trimester included an irregular placental-myometrial interface, anechoic placental areas, low implantation of the gestational sac, and placenta previa and may be detected early in gestation. In our case, these features were visualized at 13 weeks and became more pronounced in the second trimester. According to Elhawary et al. [22], the sensitivity and specificity of ultrasound to detect placenta accreta are 82.0% and 89.6%, respectively, while the positive and negative predictive values were 72.7% and 92.8% despite a relatively small sample size (*n* = 39). However, Dwyer et al. [3] had a similar sample size (*n* = 32) and reported 93% sensitivity and 71% specificity in identifying placenta accreta. Given the evidence, we believe that screening and diagnosing placenta accreta in high-risk populations are possible with the use of ultrasound imaging in the first trimester.

**Figure 2:**
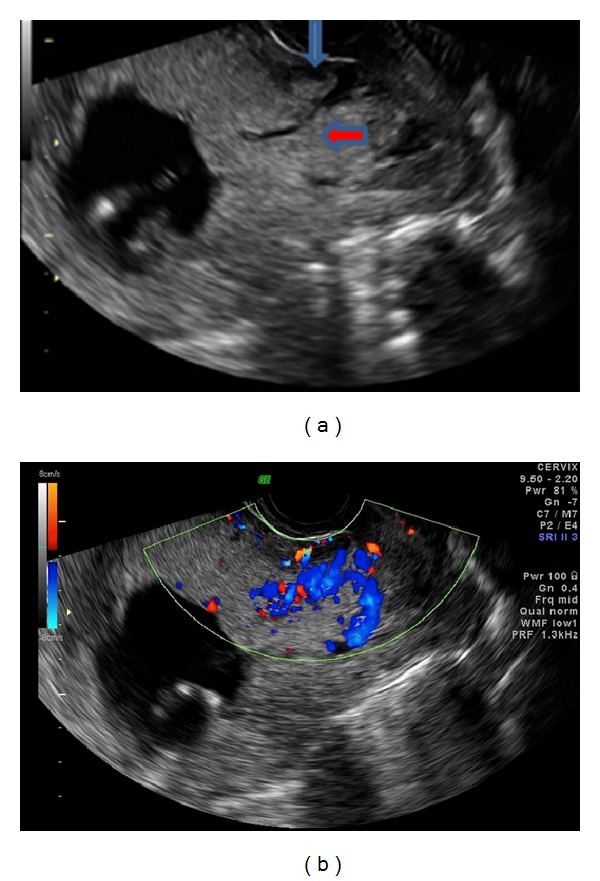
(a) Transvaginal scan at 13 weeks' gestation demonstrating placental lacunae (red arrow) and loss of placental-myometrial interface (blue arrow). (b) Colour Doppler demonstration of vascular placental lacunae with turbulent flow.

In addition, the use of color Doppler may significantly improve the findings observed with grayscale ultrasound by enhancing the ability to identify the vascular anatomy of the placenta. In cases of placenta accreta, increased vascularization of the placental-myometrial interface is an indication of an abnormality [23]. According to Elhawary et al. [22], an abnormal color Doppler imaging pattern is a helpful finding for identification of placenta accreta. The most common findings identified by color Doppler are placental lacunae with turbulent blood flow and a hypervascular serosa-bladder interface. Similarly, these features were observed in our case (Figure 2). In addition, colour Doppler sonography showed high sensitivity (82%) and specificity (89.6%) while the positive predictive and negative predictive values were 72.7% and 92.8%, respectively. These findings align with Levine et al. [24] who reported similar results with color Doppler imaging (sensitivity and specificity 86% and 92%, resp.), thus adding further support for this diagnostic technique.

While allowing imaging of vascular anatomy using multiplanar views, three-dimensional sonography may be helpful in identification of placenta accreta but will likely not replace highly accurate traditional imaging methods for screening of this condition. Similarly, magnetic resonance imaging (MRI), due to limitations of access, will likely not usurp the role of sonography in screening and diagnosis of placenta accreta despite high sensitivities (80–88.8%) and specificities (65–86.6%) [3, 22, 25]. Imaging features on MRI consistent with placenta accreta include the presence of dark intraplacental bands on T2 weighted images, heterogeneous placental signal, uterine wall bulging, and focal interruptions in the myometrial wall [22]. However, MRI may be useful adjunct in uncertain cases and assist with differentiation and elimination of other implantation anomalies including placenta percreta and increta.

While screening for abnormal placental location such as placenta previa is integral to routine second trimester sonography, screening for abnormal placental attachment found in placenta accreta is not and hence diagnosis of accreta may be either made late in gestation when morphological appearance is more pronounced or missed altogether and accomplished intrapartum when the placenta fails to separate. With the adoption of first trimester aneuploidy screening a window of opportunity has opened up to facilitate early diagnosis of this morbid condition. A high index of suspicion due to identifiable risk factors and clinical presentation along with close attention to placental morphology will facilitate early diagnosis. The advantages of early diagnosis include improved parental counseling with increased management options that include early termination of pregnancy. In our case, the patient presented with risk factors including previous Caesarean section and placenta previa in addition to abnormal bleeding. Consequently, this produced a high index of suspicion of a diagnosis of placenta accreta which was evident on close ultrasound examination of the first trimester placenta. The parents were counseled extensively regarding their options, including interruption of the pregnancy due to the high morbidity and mortality risks. This option would not have been readily available to them had the diagnosis been made in later pregnancy, after the point of fetal viability. The parents chose not to exercise their option to terminate the pregnancy and explored the possibility of conservative management before choosing elective Caesarean hysterectomy.

## 4. Conclusion

Over the last several decades, increasing rates of placenta previa and accreta have mirrored rapidly rising rates of Caesarean section. It has been estimated that if these trends in operative abdominal delivery continue to rise unabated, by 2020 Cesarean section rates in the United States will approximate 56% and be accompanied by an extra 4504 cases of placenta accreta leading to an additional 130 maternal deaths annually [26]. As obstetric imagers performing first trimester ultrasound, we have a privileged view into the early intrauterine environment. Diligent examination of the placental morphology to rule out placenta accreta, especially in high-risk patients, should be encouraged to improve patient education and choice and limit morbidity of this rising iatrogenic placental disorder.
